# A Case of Disseminated Histoplasmosis From California, in the Setting of Secondary Hemophagocytic Lymphohistiocytosis: A Diagnostic Challenge

**DOI:** 10.1177/23247096231156007

**Published:** 2023-02-17

**Authors:** Ameish Govindarajan, Rowis Sous, Frederick Venter, Tyler Torrico, Natalie Karapetians, Arash Heidari, Everardo Cobos, Greti Petersen

**Affiliations:** 1 Department of Medicine, Kern Medical UCLA, Bakersfield, USA; 2 Department of Psychiatry, Kern Medical UCLA, Bakersfield, USA; 3 Division of Infectious Diseases, Department of Medicine, Kern Medical UCLA, Bakersfield, USA

**Keywords:** *Histoplasma capsulatum*, hemophagocytic lymphohistiocytosis, bilateral adrenal masses, fungus, dissemination, infectious disease

## Abstract

*Histoplasma capsulatum* is a geographically specific dimorphic fungus that can cause a spectrum of diseases. While most cases are asymptomatic pulmonary infections, in severe cases, particularly in immunocompromised patients, disseminated disease can occur. Histoplasmosis in California is limited to only a few case reports. In this article, we describe a rare case of disseminated histoplasmosis in a non-endemic region presenting with diagnostically challenging symptomatology, including altered mental status, status epilepticus, septic shock, and bilateral adrenal masses.

## Introduction

*Histoplasma capsulatum* is a geographically specific dimorphic fungus that causes histoplasmosis (HP). In America, this fungus is most prevalent in the Eastern United States, specifically in the Ohio and Mississippi River Valleys.^1^ Rarely have cases and outbreaks been reported in non-endemic areas. Although California is highly endemic for another dimorphic fungus, *Coccidioides*, which causes coccidioidomycosis, HP is rarely reported. Three cases were reported in San Diego in 1966,^2^ 2 cats in Central California were diagnosed with HP in 2004,^3^ and 1 case of disseminated HP in an AIDS patient was reported in southern California in 2019.^4^

Nonetheless, there has been an increasing concern of occupational exposures to HP. In 2001, a large outbreak of HP occurred after a group of students traveled to the non-endemic region of Mexico where a hotel under construction was determined to be the common risk factor for the outbreak.^5^ Similarly, in 2008, another outbreak was reported after a group traveled to El Salvador to renovate a church.^6^ In 2013, another outbreak was associated with the renovation of an old house in Quebec, Canada.^7^ In this article, we aim to highlight the importance of retaining HP in the differential diagnosis while working up patients with similar presentations even in non-endemic states as we describe another case of HP in a patient from California whose only known risk factor was occupational exposure from years prior and military service in Germany when he was 18 years and in Texas when he was 21 years old. This case report highlights the importance of obtaining a thorough social history, including previous residence, travel, and occupational exposures.

## Purpose of Study

To present a rare case of a *Histoplasma capsulatum* in a patient presenting with altered mental status and bilateral adrenal masses in a non-endemic region.

## Methods

Case report and literature search.

## Case Presentation

**Figure 1.**
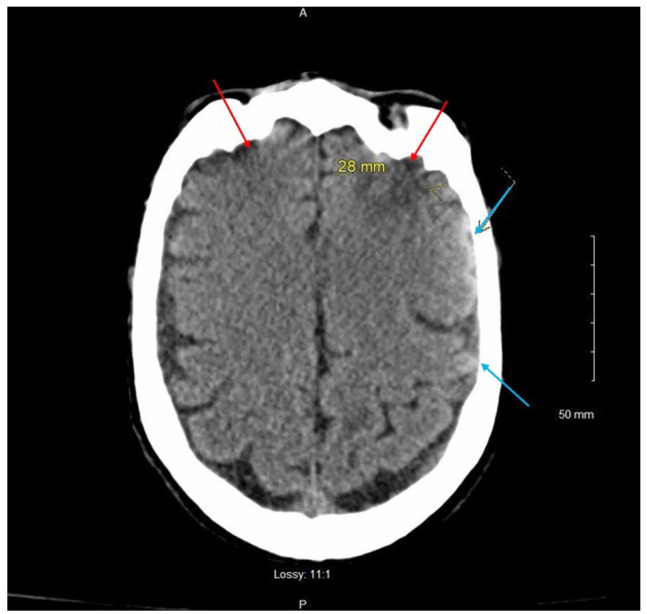
Magnetic resonance imaging (MRI) of the brain demonstrating bifrontal hemorrhagic contusions (red arrows, larger on the left), scattered subarachnoid hemorrhages without demonstrable mass effect or midline shift (blue arrows), and few scattered tiny hypointense areas on gradient echo.

A 58-year-old Caucasian male with a medical history of hypertension, remote history of coccidioidomycosis, and tobacco use presented with acute-onset altered mental status and 70-pound unintentional weight loss over the previous 6 months. The patient was found to be in status epilepticus refractory to anti-epileptic drugs secondary to traumatic brain injury (TBI) from multiple ground-level falls, and magnetic resonance imaging (MRI) of the brain demonstrated bifrontal hemorrhagic contusions (larger on the left), scattered subarachnoid hemorrhages without demonstrable mass effect or midline shift, and few scattered tiny hypointense areas on gradient echo (Figure 1). Subsequent magnetic resonance angiography (MRA) showed no demonstrable intracranial aneurysm or occlusive vascular disease in the anterior or posterior circulations.

**Figure 2.**
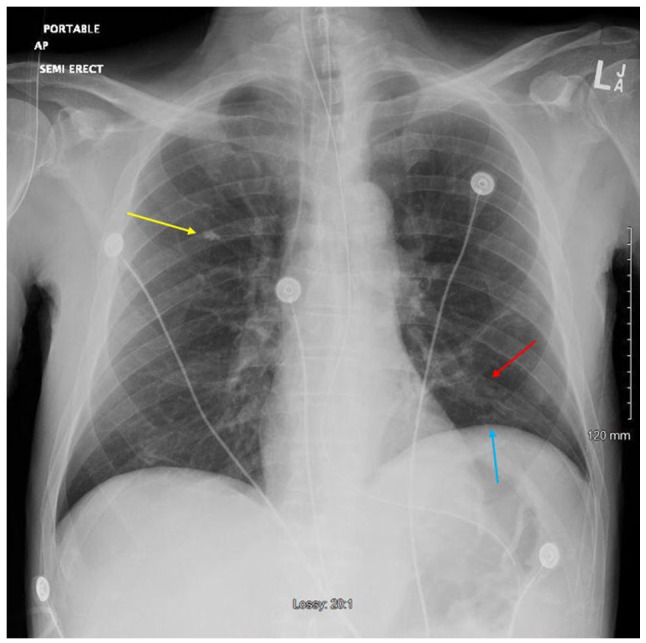
Chest x-ray taken on admission showing left basilar atelectasis (red arrow), left hemi diaphragm elevation (blue arrow), and a right upper lobe irregular calcification (yellow arrow).

The patient went into septic shock, presumably due to aspiration pneumonia and urinary tract infection, requiring vasopressors and intravenous antibiotics. He was intubated and transferred to the intensive care unit for acute respiratory failure, severe anion gap metabolic acidosis with pH 6.9, HCO_3_ 8 mmol/L, and a corrected anion gap of 22 mmol/L, acute renal failure with a creatinine of 14.8 mg/dL, and hyperkalemia of 6.6 mmol/L (Table 1), and started on hemodialysis. Initial chest x-ray revealed left basilar atelectasis, left hemidiaphragm elevation, and a right upper lobe irregular calcification (Figure 2).

**Table 1.** Complete Laboratory and Immunology Work Up.

Complete blood count w/ differential		Immunology	
WBC	**2.2 × 10**^9^ **/L**	SARS-CoV-2 RNA	Negative
ANC	**1.9 × 10**^9^ **/L**	Hepatitis B sAg	Non-reactive
Hb	**7.9 g/dL**	Hepatitis C Ab	Non-reactive
HCT	**23.6%**	QuantiFERON-TB	Negative
MCV	82.6 fL	HIV Ag/Ab screening	Non-reactive
PLT	**66 × 10**^9^ **/L**	HIV 1 RNA PCR	Not detected
Iron studies		Syphilis Ab	Non-reactive
Iron	**15 µmol/L**	Coccidioidomycosis IgM	**Weakly reactive**
TIBC	**130 µmol/L**	Coccidioidomycosis IgG	Non-reactive
Ferritin	**1186 µg/L**	Complement fixation	<1:2
Coagulation studies		Histoplasma Ab	**Detected**
PT	**16 seconds**	Histoplasma yeast phase Ab	**1:8**
PTT	**41.5 seconds**	Histoplasma Ag, adrenal gland	**Positive**
INR	**1.28**	Histoplasma Ag, urine	**Positive**
Complete metabolic panel		Cerebrospinal fluid	
Na	142 mEq/L	Opening pressure	19 cm H_2_O
K	3.5 mEq/L	WBC	4 cells
Cl	**111 mEq/L**	RBC	**55 cells**
CO_2_	20 mEq/L	Glucose	**29 mmol/L**
Ca	8.2 mg/dL	Protein	**66 mg/L**
BUN	**52 mg/dL**	Coccidioidomycosis IgM	Non-reactive
Cr	**6.41 mg/dL**	Coccidioidomycosis IgG	Non-reactive
Glu	80 mg/dL	Complement fixation	<1:1
eGFR	**9 mL/min/1.73**	Histoplasma Ag	Negative
Albumin	**1.5 g/dL**	Endocrinology	
ALP	**260 IU/L**	Cortisol AM	23.1 µg/dL
ALT	21 IU/L	Aldosterone	**1 ng/dL**
AST	34 IU/L	Plasma renin activity	0.47
Total bilirubin	**1.6 mg/dL**	Aldosterone/renin ratio	2.1
TSH	1.911 mIU/L	Urine free cortisol	16.8 µg/24 h
Microbiology			
Blood cultures	No growth		
Blood fungal cultures	No growth		
CSF fungal cultures	No growth		

Abbreviations: WBC, white blood cell; SARS-CoV-2, severe acute respiratory syndrome coronavirus 2; ANC, absolute neutrophil count; Hb, hemoglobin; HCT, hematocrit; MCV, mean corpuscular volume; PLT, platelet; PCR, polymerase chain reaction; TIBC, total iron-binding capacity; PT, prothrombin time; PTT, partial thromboplastin time; INR, international normalized ratio; RBC, red blood cell; BUN, blood urea nitrogen; eGFR, estimated glomerular filtration rate; ALP, alkaline phosphatase; ALT, alanine transaminase; AST, aspartate transaminase; TSH, thyroid-stimulating hormone; CSF, cerebrospinal fluid. Bold values are the positive findings.

**Figure 3.**
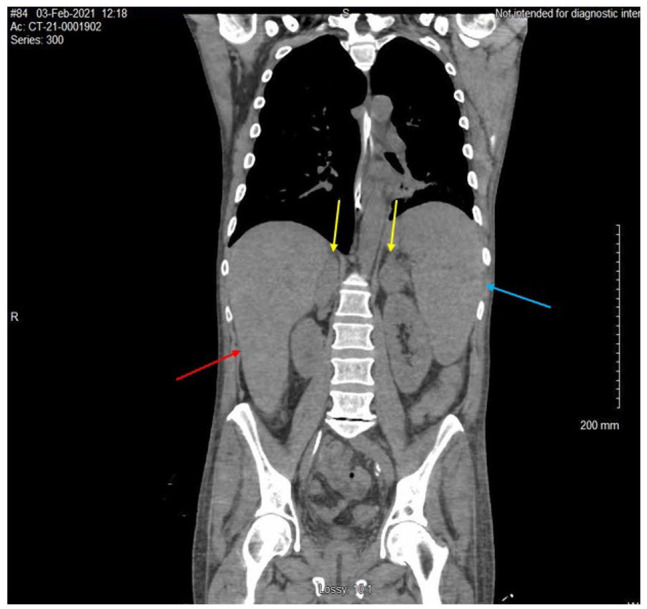
Computed tomography (CT) without contrast of the abdomen and pelvis revealed hepatomegaly with a craniocaudal length of 21 cm (red arrow), splenomegaly with a length of 18.2 cm (blue arrow), bilateral adrenal masses measuring 4 cm each (yellow arrow), and enlarged pericaval lymph nodes with a length of 2.3 cm × 1.7 cm × 4 cm.

**Figure 4.**
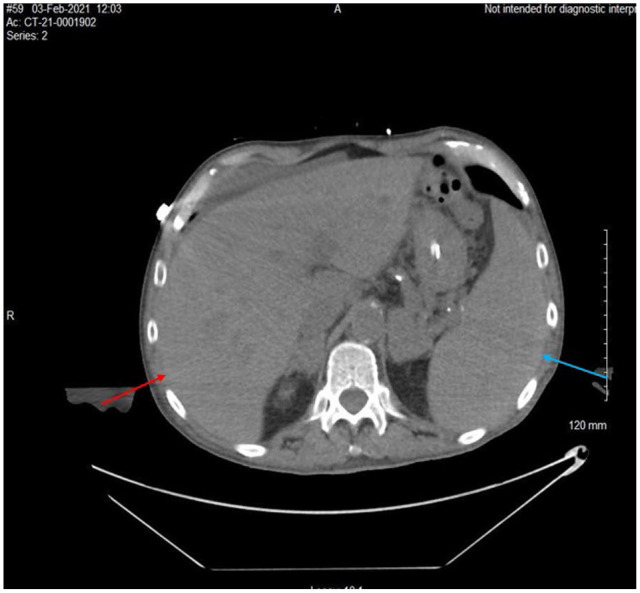
Computed tomography (CT) without contrast of the abdomen and pelvis revealed hepatomegaly with a craniocaudal length of 21 cm (red arrow), splenomegaly with a length of 18.2 cm (blue arrow), bilateral adrenal masses measuring 4 cm each, and enlarged pericaval lymph nodes with a length of 2.3 cm × 1.7 cm × 4 cm.

The patient was also found to have pancytopenia and required multiple packed red blood cell and platelet transfusions. Given this along with recent weight loss, the team performed cancer workup that included a computed tomography (CT) without contrast of the abdomen and pelvis, which revealed hepatomegaly with a craniocaudal length of 21 cm, splenomegaly with a length of 18.2 cm, bilateral adrenal masses measuring 4 cm each, and enlarged pericaval lymph nodes with a length of 2.3 cm × 1.7 cm × 4 cm (Figures 3 and 4). Adrenal insufficiency was ruled out with morning cortisol of 23.1 µg/dL. A transthoracic echogram revealed no evidence of heart or valvular disease.

Despite empiric antibiotics throughout the hospital course, the patient continued to maintain low-grade fevers. Initial laboratory values noted to have an elevated ferritin 1186 µg/L, and the patient remained transfusion dependent due to pancytopenias with hemoglobin 7.9 g/dL and platelets 66 × 10^9^/L. Splenomegaly was seen on CT scan, raising the specter of possible hemophagocytic lymphohistiocytosis (HLH) in the setting of potential malignancy or possible infection.

All cultures, including fungal cultures, from cerebrospinal fluid (CSF), blood, and urine had no growth. Laboratory results were negative for HIV, hepatitis B and C, syphilis, and QuantiFERON-TB Gold. Given the patient’s history of coccidioidomycosis infection living in an endemic area, serology was ordered and showed immunodiffusion IgM weakly reactive and immunodiffusion IgG non-reactive, with complement fixation <1:2. A lumbar puncture was performed, which ruled out meningitis with an opening pressure of 19 cm H_2_O, white blood cell (WBC) count of 4 cells/µL, red blood cell (RBC) count of 55 cells/µL, glucose level of 55 mg/dL, and protein level of 66 mg/dL.

Further collateral information was obtained from the patient’s family who reported that the patient had a productive cough and a 2-week history of dyspnea, suffered multiple ground-level falls, and had poor oral intake. The patient was working as an independent handyman and school maintenance worker. Much of his job entailed working with plumbing in attics, basements, underneath sinks, and sewer lines. He denied exploring caves or being involved with birds/bats. His travel history included military service: he was stationed in Germany when he was 18 years old and in Texas when he was 21 years old.

Due to suspicion of possible malignancy with pancytopenia, significant weight loss, and hepatosplenomegaly, a CT-guided bone marrow biopsy was performed. Although it was suboptimal due to a lack of adequate core, the biopsy revealed a normocellular sample with unremarkable trilineage hematopoiesis; no evidence of hemophagocytosis, lymphoid aggregates, or metastatic malignancy; and no evidence of fungal elements by Grocott’s methenamine silver (GMS) stain.

**Figure 5.**
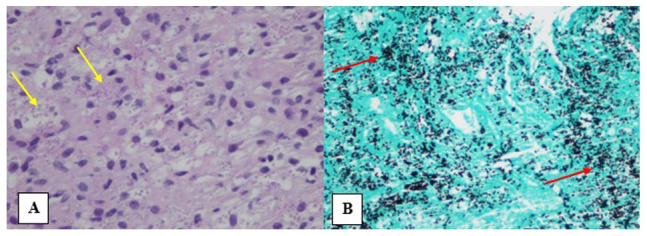
Left adrenal biopsy stained with: (A) histoplasmosis on hematoxylin and eosin. Hematoxylin and eosin demonstrated small, round, shadowlike microorganisms (yellow arrow). (B) Histoplasmosis stained with Grocott’s methenamine silver. Grocott’s methenamine silver stained the microorganisms black (red arrow).

Subsequently, a left adrenal biopsy was performed and stained with hematoxylin and eosin, demonstrating small, round, shadowlike microorganisms that turned dark black when stained with GMS—consistent with HP (Figure 5). Serological tests revealed positive histoplasma antibody, with yeast phase antibody titer of 1:8, positive urine histoplasma antigen, and positive adrenal gland histoplasma antigen. At this point, the diagnosis of disseminated HP was made.

The patient was started on liposomal amphotericin B therapy for disseminated HP with improvement of mental and functional status, participating with physical therapy and was transferred to the Veterans Affairs hospital in the region to complete his treatment course.

## Discussion

HP infection typically results from inhalation of *Histoplasma* spores from soils contaminated by bird and bat droppings.^8^ These spores settle in the alveolar spaces, and once in the human body, the fungus transforms into its yeast form.^9^ The immune system responds with alveolar macrophage phagocytosis of the yeast. It is within these WBCs that the fungus begins to replicate, as these macrophages travel throughout the human body via the reticuloendothelial system.^9^ This mechanism for fungal dissemination allows for infection of various organ systems, the most common of which is the adrenal glands.^10,11^ This progressive dissemination occurs when the immune system is unable to formulate a sufficient T-cell-mediated response to clear the yeast from the human body Patients are often unaware of contracting the fungus, given that the majority of cases are asymptomatic.^8^ Nonetheless, symptomatic HP has 3 forms of presentation: acute primary HP, chronic cavitary HP, and progressive disseminated HP.^12^ Disseminated HP may present years after the initial infection, and it may be the primary presentation if the initial infection was asymptomatic.^13^ Although rare, the risk for disseminated HP is more probable in immunocompromised patients.^9^

Based on the HLH-2004 criteria, to make the diagnosis of HLH requires 5 out of 8 of the following: fever, splenomegaly, peripheral cytopenias in at least 2 lines, high triglycerides or low fibrinogen, ferritin levels over 500 µg/L, high-soluble interleukin (IL)-2 receptor (CD25), and no natural killer cell activity. Our patient met 5 of these criteria with fevers, splenomegaly seen on CT, cytopenias with hemoglobin 7.9 g/dL and platelets 66 × 10^9^/L, ferritin 1186 µg/L, and detected soluble IL-2 receptor α (CD25) 23 518 pg/mL, thus confirming the diagnosis of secondary HLH.

In the current literature, there has been approximately 26 cases of secondary HLH in the setting of disseminated HP, and within most of these cases, the patients were noted to be immunocompromised. HLH is caused by abnormal activation of macrophages and lymphocytes, which results in inflammation, tissue damage, and is life threatening. HLH is typically seen in infants; however, it can be diagnosed in adults as old as 70 years of age, with a higher predisposition seen in males, such as our patient. Successful treatment of HLH is often thwarted due to a delay in diagnosis. These delays can be caused by varied clinical presentations and overall rarity of the disease in the concomitant setting of another disease.

The diagnosis of HP in non-endemic regions is often delayed and difficult to make. This case report highlights the importance of obtaining a thorough social history, including previous residence, travel, and occupational exposures. In addition, findings such as bilateral adrenal lesions in the adult patient should raise suspicion to broaden differential diagnosis to include atypical infectious etiology. The patient’s current residence in a geographical location highly endemic to coccidioidomycosis, which is caused by another dimorphic fungus, complicated the HP diagnosis due to similarities between the 2 fungal infections. Furthermore, the patients’ symptoms of altered mental status and associated weight loss are common to disseminated coccidioidal infection, which is endemic to the California San Joaquin Valley where our patient was born and resided in for a majority of his life. He worked as handyman and school maintenance worker, often immersed in confined humid spaces: attics, sinks, water pipes, and sewer lines. Additional differential diagnoses also included Whipple’s disease due to extensive and prolonged exposure to sewage and weight loss; however, there was no diarrhea or joint involvement.

The clinicians reasoned that the sudden 70-pound weight loss was caused by poor oral intake which also led to hypotension and episodes of hypoglycemia with subsequent ground-level falls. Resultant TBI caused structural abnormalities seen on MRI of the brain (Figure 2). TBI superimposed on metabolic encephalopathy is believed to have caused the focal status epilepticus seen in the Emergency Department.

On further investigation, it was found that his military service led him to travel to Germany for 2 years when he was 18 years old and then to Fort Hood in Texas for another year after that. HP has been described in both geographical regions.^9,14,15^ Most cases of HP diagnosed in California are attributed to fungal exposure outside of the state, often through previous residence or travel, similar to this case.^16^ Both are contracted through the inhalation of fungal spores.^17^ The gold standard workup for diagnosis of disseminated HP to adrenal glands is ultrasound-guided fine needle aspiration and cytology (USG-FNAC). This usually follows a discovered localized suspicious focal area, best revealed by CT. Disseminated HP to the adrenals may be found as enlargement of the adrenals with central hypodensity, suggesting gland destruction along with enhancement of the internal septations and peripheral rim.^13^ Microscopic examination of the aspirate may reveal caseous granuloma and fungal elements, including hyaline granules and intracellular or extracellular single buds with narrow bases.^18^ The growth of *Histoplasma* is confirmatory. For our patient, abdominal and pelvic CT revealed hepatosplenomegaly, enlarged pericaval lymph nodes, and bilateral adrenal masses. USG-FNAC of the left adrenal demonstrated small, round, shadowlike microorganisms.

Infectious Disease Society of America (IDSA) recommends treating moderate to severe cases of HP with liposomal amphotericin B with the transition to itraconazole. In mild cases, treatment can be achieved with itraconazole alone.^19^ Management requires up to 12 months of pharmacological treatment with monitoring of adrenal function. Our patient was initiated on liposomal amphotericin B, given his severe presentation, and showed improvement with plans of transitioning to itraconazole in the near future.

## Conclusion

Diagnosing HP in individuals in non-endemic regions is challenging and mostly delayed. The exposure history of the endemic area is usually found after the diagnosis is already made by other means such as histopathology, serology, or cultures. Clinicians must obtain a thorough social history when reactivation of dormant infections is suspected.
